# Determining the culturability of the rumen bacterial microbiome

**DOI:** 10.1111/1751-7915.12141

**Published:** 2014-07-01

**Authors:** Christopher J Creevey, William J Kelly, Gemma Henderson, Sinead C Leahy

**Affiliations:** 1 Animal and Bioscience Research Department, Animal and Grassland Research and Innovation Centre, TeagascGrange, Dunsany, Co. Meath, Ireland; 2 Institute of Biological, Environmental and Rural Sciences, Aberystwyth UniversityAberystwyth, Ceredigion, UK; 3 Grasslands Research Centre, AgResearch LimitedPalmerston North, New Zealand; 4 New Zealand Agricultural Greenhouse Gas Research CentrePalmerston North, New Zealand

## Abstract

The goal of the Hungate1000 project is to generate a reference set of rumen microbial genome sequences. Toward this goal we have carried out a meta-analysis using information from culture collections, scientific literature, and the NCBI and RDP databases and linked this with a comparative study of several rumen 16S rRNA gene-based surveys. In this way we have attempted to capture a snapshot of rumen bacterial diversity to examine the culturable fraction of the rumen bacterial microbiome. Our analyses have revealed that for cultured rumen bacteria, there are many genera without a reference genome sequence. Our examination of culture-independent studies highlights that there are few novel but many uncultured taxa within the rumen bacterial microbiome. Taken together these results have allowed us to compile a list of cultured rumen isolates that are representative of abundant, novel and core bacterial species in the rumen. In addition, we have identified taxa, particularly within the phylum *Bacteroidetes*, where further cultivation efforts are clearly required.

This information is being used to guide the isolation efforts and selection of bacteria from the rumen microbiota for sequencing through the Hungate1000.

## Introduction

Ruminants have evolved a symbiotic relationship with a complex microbiome consisting of bacteria, archaea, fungi, protozoa, and viruses located in their fore-stomach (reticulorumen) that allows these animals to utilize the lignocellulose component of plant material as their main energy source. The microbial degradation of lignocellulose, and fermentation of the released soluble sugars, produces short-chain fatty acids that are absorbed across the rumen epithelium and utilized by the ruminant for growth, while the microbial cells pass from the rumen to the digestive tract where they become the main source of amino acids and protein for the animal. Other fermentation end-products, including hydrogen, carbon dioxide, formate and methyl-containing compounds, are important substrates for methanogenesis. Consequently, rumen microbes are central to the optimal digestive functioning of the animal, and the lack of sufficient understanding of the rumen microbiome is one of the major knowledge gaps hindering effective enhancement and modification of rumen function (Kingston-Smith *et al*., 2013).

Bacteria are the most numerous organisms in the rumen microbiome, being present at 10^10^ to 10^11^ g^−1^ of content and making up more than 50% of the cell mass. Studies of rumen bacteria began with the development of techniques for cultivating strictly anaerobic organisms and have attempted to answer three questions: ‘Who is there?’, ‘How many?’ and ‘What are they doing? As a consequence, most of the knowledge we have of rumen bacteria has arisen from traditional microbiological methods involving the isolation and cultivation of pure strains and species. Microscopic examination of rumen contents shows that the rumen contains a rich diversity of microbes, and many different bacteria have been isolated from this environment. Some, such as *Lampropedia* and *Quinella,* are distinctive enough to be identified by morphology alone (Clarke, 1979), but most have been described on the basis of their metabolism and probable functional role in the rumen. Many of the best characterized rumen bacteria were described in the pioneering studies of rumen microbiology which isolated representatives of the functionally significant bacterial groups (Bryant, 1959; Hungate, 1966). Knowledge of rumen bacterial diversity has increased in subsequent years as additional genera of rumen bacteria have been isolated and characterized. These culture-based studies of bacterial morphology, physiology and metabolism have revealed a bacterial community that includes both generalists and specialists able to convert large plant polymers into a variety of smaller organic compounds.

However, in recent years, emphasis on bacterial culturing has been reduced and our knowledge of the rumen bacteria across different diets, ruminant species and geographical locations has quickly expanded through the insights of culture-independent methods, such as 16S rRNA gene surveys aimed at classifying and quantifying the microbes present (Brulc *et al*., 2009; Kong *et al*., 2010; Pitta *et al*., 2010; De Menezes *et al*., 2011; Hess *et al*., 2011; Kim *et al*., 2011; Li *et al*., 2011; Fouts *et al*., 2012; Jami and Mizrahi, 2012; Kav *et al*., 2012; Pope *et al*., 2012; Wu *et al*., 2012). These studies are providing new answers to the questions ‘Who is there?’ and ‘How many?’, and it is clear that the number of rumen cultured representatives has not kept pace with this expansion in knowledge. While 16S rRNA gene sequences are useful to take a census of bacteria present in the community, they provide only very limited functional genetic information and thus do not address the question of ‘What are they doing?’ As a result, genome sequencing has now become a starting point for analyses that reveal a microbe’s metabolic potential. However, cultivation and isolation of strains remain a challenge, and there is a need for physiological studies to verify predictions based on genome sequence data.

The Hungate1000 project (www.Hungate1000.org.nz) seeks to produce a reference set of rumen microbial genome sequences from cultivated rumen bacteria and archaea, together with representative cultures of rumen anaerobic fungi and ciliate protozoa. The resulting data will enable genome-based research aimed at understanding rumen function, feed conversion efficiency, methanogenesis and plant cell wall degradation. It will underpin the analysis and comprehension of rumen metagenomic and metatranscriptomic sequence datasets and it will broaden our knowledge of rumen microbial phylogenetic diversity through genome sequencing of novel microorganisms. To guide the isolation efforts and selection of bacteria from the rumen microbiota for sequencing through the Hungate1000, we performed a meta-analysis of available and comparable rumen 16S rRNA-based studies overlaid with cultured representative data. This was done not only to assess the diversity present and to identify cultured taxa that remain unrepresented in reference genome collections, but also to identify taxa that require more cultivation effort. The resulting list of taxa is currently being used by the Hungate1000 for sequencing of previously unsequenced organisms found in association with ruminants. It is hoped this list of taxa will serve as a resource for the rumen microbiology community interested in the isolation of novel members of the microbiome. Sequencing of these organisms will bring us closer to completing a comprehensive reference genome collection for the rumen microbiome.

## Results and discussion

### Rumen bacteria in culture

Using information from culture collections, scientific literature, the National Center for Biotechnology Information (NCBI) and Ribosomal Database Project (RDP, Cole *et al*., 2014) databases we identified cultivated rumen bacteria, which include members of 88 different bacterial genera belonging to nine phyla (Table 1 and Supporting Information Table S1). A survey of five international culture collections (American Type Culture Collection (ATCC), Culture Collection University of Göteborg (CCUG), Leibniz Institute DSMZ-German Collection of Microorganisms and Cell Culture, Japan Collection of Microorganisms (JCM), The Belgian Co-ordinated Collections of Micro-organisms (BCCM/LMG) gave 146 bacterial cultures of rumen origin (Supporting Information Table S2), but members of only seven phyla. These collections are dominated by members of the phyla *Firmicutes*, *Proteobacteria* and *Actinobacteria*, which together make up 90% of the cultures listed. Members of the *Firmicutes* and the family *Lachnospiraceae* in particular, appear more amenable to laboratory culture, as there are 45 genera of *Firmicutes* that have been cultured from the rumen. In contrast, it is notable that the phylum *Bacteriodetes* is represented by just five isolates belonging to two genera. While these culture collection isolates cover all the major taxonomic groups and include several well-described organisms that have long been known to have key roles in rumen function, they clearly do not represent the full diversity of the rumen microbiome. There are several cultured bacteria that have yet to be characterized and named as some research groups have taken up the challenge to bring additional rumen organisms into cultivation (Koike *et al*., 2010; Kenters *et al*., 2011; Noel, 2013; Nyonyo *et al*., 2013). A recent study has focused on isolating bovine hindgut bacteria through an enrichment approach using bovine faeces and the bacterial complement of this environment is similar to, but different from, that of the rumen (Ziemer, 2014). In addition, the taxonomy of rumen bacteria is still poorly defined and genera such as *Eubacterium* and *Prevotella* contain groups of organisms that are only distantly related to one another, while new genus assignments have been proposed for many *Clostridium* species (Yutin and Galperin, 2013). Several rumen bacteria belong in these proposed new genera (*Erysipelatoclostridium*, *Lachnoclostridium*, *Peptoclostridium* and *Ruminiclostridium*). Of the 88 bacterial genera identified in this analysis, 20 have had at least one genome sequencing project completed. Several more are reported as being targeted (http://www.genomesonline.org/) or in progress by community sequencing programmes such as the Genomic Encyclopedia of Bacteria and Archaea (GEBA, Wu *et al*., 2009) however, many organisms remain to be sequenced in order to take full advantage of the current available cultured isolates (Supporting Information Table S1).

**Table 1:** Comparison of rumen culture information against 16S rRNA gene based studies examined

Phylum	Cultured genera *n* (%)	Cultured isolates^a^ *n* (%)	Kim *et al*., 2011 study *n* (%)	This study *n* (%)
*Actinobacteria*	11 (13)	25 (17)	107 (1)	41 (2)
*Bacteriodetes*	6 (7)	5 (3)	3605 (27)	907 (38)
*Cyanobacteria*	–	–	1 (0)	3 (0)
*Fibrobacteres*	1 (1)	7 (5)	112 (1)	16 (1)
*Firmicutes*	45 (51)	90 (62)	7797 (58)	1263 (53)
*Fusobacteria*	1 (1)	–	10 (0)	1 (0)
*Planctomycetes*	–	–	17 (0)	1 (0)
*Proteobacteria*	20 (23)	16 (11)	928 (7)	64 (3)
*Spirochaetes*	1 (1)	2 (1)	144 (1)	48 (2)
*Synergistetes*	1 (1)	1 (1)	382 (3)	11 (0)
*Acidobacteria*	–	–	1 (0)	3 (0)
*Tenericutes*	2 (2)	–	16 (0)	21 (1)
*Chloroflexi*	–	–	14 (0)	4 (0)
*Deferribacteres*	–	–	4 (0)	0 (0)
*Lentisphaerae*	–	–	12 (0)	2 (0)
*Verrucomicrobia*	–	–	57 (0)	1 (0)
OP10	–	–	1 (0)	0 (0)
SR1	–	–	32 (0)	4 (0)
TM7	–	–	39 (0)	14 (1)
*Nitrospira*	–	–	–	1 (0)

a Survey of five international culture collections – American Type Culture Collection (ATCC), Culture Collection University of Göteborg (CCUG), Leibniz Institute DSMZ-German Collection of Microorganisms and Cell Culture, Japan Collection of Microorganisms (JCM), The Belgian Co-ordinated Collections of Micro-organisms (BCCM/LMG). Cultured genera see Supporting Information Table S1, Cultured Isolates see Supporting Information Table S2.

### Culture-independent analysis of rumen bacteria

With the development of culture-independent methods and the observation that only a fraction of the range of bacteria found in the rumen existed in culture, we sought to further assess the diversity and culturability of the rumen bacterial community through a meta-analysis of 16S rRNA gene based surveys. Seven datasets that spanned, at a minimum, the V1 and V2 regions were chosen for inclusion in the analysis. On average 17% of the sequences were discarded from each dataset after cleaning and filtering, and on average were reduced in length by 20% (Supporting Information Table S3). After dataset specific clustering, the number of operational taxonomic units (OTUs) per dataset ranged from 520 (Brulc *et al*., 2009) to 2691 (Pitta *et al*., 2010) reflecting the number of reads sequenced in each study (2811 to 1 166 013 respectively). These are referred to as the *de novo* datasets in the following sections.

To determine the culturable aspect of this study, 187 236 sequences representing all bacterial isolates identified from any environment were downloaded from the RDP database. After clustering at 97%, 15 628 sequences representing each of the clusters were retained for further analyses. The OTUs from each of the *de novo* datasets and the selected RDP sequences were combined into a dataset containing 25 003 sequences. When clustered together they resulted in the identification of 22 031 universal OTUs (defined as all bacterial OTUs found from any molecular survey, regardless of environmental origin), of which there were rumen representatives in 2405. We compared our results with a previous study of the rumen microbiome (Kim *et al*., 2011). Both studies show a similar predominance of the phyla *Bacteriodetes* and *Firmicutes*, 85% and 91% respectively (Table 1). Our analysis revealed a higher portion of the phylum *Bacteriodetes* (37% versus 27%). Overall, a total of 20 bacterial phyla have been detected in the rumen, but the majority of these are rare and only nine phyla have cultured representatives.

**Figure 1:**
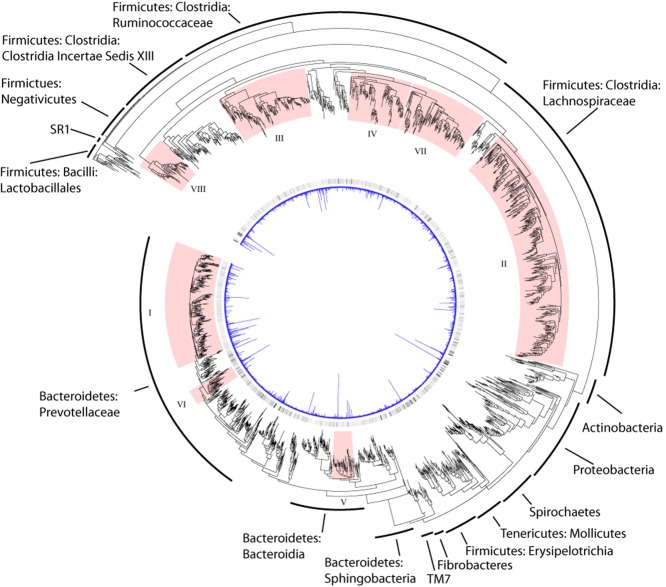
Inverted circular phylogenetic tree of the 2405 rumen bacterial OTUs identified as part of this analysis. The blue graph in the middle represents the average scaled proportion of each OTU from across the seven datasets analysed. The colour gradient surrounding that represents the prevalence of each OTU across all seven datasets analysed (dark = most prevalent, light = least prevalent). The major groups of bacteria that are represented in the tree are indicated. The clades that are most abundant in the rumen are indicated in red and numbered I to VIII in order of abundance. The statistics associated with clades numbered I to VIII are detailed in Supporting Information Table S4.

**Figure 2:**
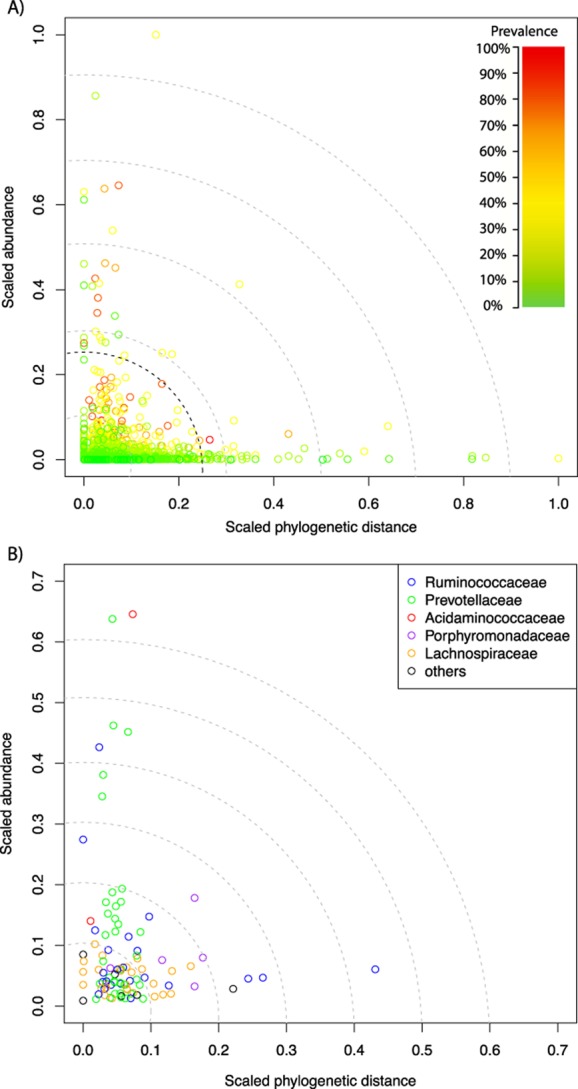
On both plots, the x-axis shows the scaled phylogenetic distance from the nearest cultured isolate and the y-axis the average scaled abundance for each OTU. A. The circles are coloured according to their prevalence across the seven datasets as indicated by the colour gradient. The black dotted line illustrates the 0.25 cut-off discussed in the text. B. The subplot of only those 98 core OTUs colour-coded according to taxonomy.

The entire 22 031 OTUs were used to create a phylogenetic tree, so tip-to-tip distances could be calculated. A subset of the tree, displayed in Fig. 1, only contains those 2405 OTUs that contained representatives from the seven *de novo* datasets analysed. The 22 031 taxa tree was manually subdivided into 300 monophyletic clades or singletons, 121 of which contained OTUs from the *de novo* datasets (Supporting Information Table S4). Our comparative analysis of these culture-independent studies highlights that there are few novel (defined as having a scaled phylogenetic distance greater than 0.25 from the nearest cultured isolate), but many uncultured taxa within the rumen bacterial OTUs identified, some of which are relatively abundant (greater than 0.25 scaled abundance) (Fig. 2A). An analysis of these OTUs suggests that organisms from the *Prevotellaceae* family dominate the rumen bacterial microbiota, followed by the families *Lachnospiraceae* and *Ruminococcaceae*, as depicted in Fig. 1. The eight most abundant clades constitute 64% of the sequences and are highlighted in Fig. 1. All belong to the phyla *Bacteroidetes* and *Firmicutes*. Clade I contains 286 OTUs and is the most abundant making up 20.6% of sequences from the dataset. This clade includes members of the family *Prevotellaceae*, but their diversity remains uncharacterized as sequences from the RDP database are represented in just six OTUs. Clade II is the second most abundant with 17% of the sequences but has a greater number of OTUs (432). This clade corresponds to bacteria from the family *Lachnospiraceae*. Other abundant *Firmicutes* are two clades of bacteria from the family *Ruminococcaceae* (Clades IV and VII), a clade containing unclassified bacteria from the order *Clostridiales* (Clade III) and a clade covering bacteria from the class *Negativicutes* (Clade VIII). For the *Bacteriodetes*, two additional clades corresponding to members of the family *Prevotellaceae* (Clade VI) and the class *Bacteroidia* (Clade V) are among the most abundant.

**Figure 3:**
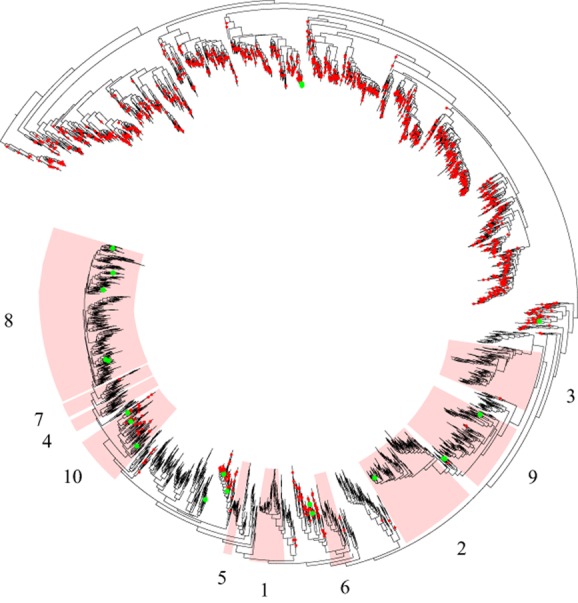
*Bacteriodetes*-specific tree of OTUs, including those from the RDP database. OTUs which contain sequences from the RDP database are marked with a red dot. The green dots highlight OTUs, which contain RDP isolates that have been annotated as of rumen origin. The statistics associated with clades numbered 1 to 10 are detailed in Supporting Information Table S4.

Almost a third of the clades (38/121), representing on average 13% of all the sequences isolated from the rumen samples and primarily from the *Bacteroidetes* phylum, contained no RDP isolate representative (Supporting Information Table S4). A *Bacteroidetes*-specific tree is illustrated in Fig. 3. This shows the 10 most abundant clades from this phylum, five of which contain no sequences from the RDP database. Members of the genus *Prevotella* have frequently been reported as being highly abundant in the rumen (Stevenson and Weimer, 2007) and have been divided into two main groups that comprise species found only in the rumen and species that cluster with *Prevotella* strains isolated from other environments (Ramšak *et al*., 2000). The rumen specialists include the species *Prevotella brevis* and *Prevotella ruminicola* and these belong in the most abundant clade (Clade 8 in Fig. 3, Supporting Information Fig. S1) of rumen bacteria identified in this study. Many of the OTUs from this clade give a best match to a cultured isolate but at low homology. The other two *Prevotella* species isolated from the rumen (*Prevotella albensis* and *Prevotella bryantii*) cluster with non-rumen strains (Clade 10 in Fig. 3, Supporting Information Fig. S1), and it has been suggested that they may also inhabit the oral cavity (Ramšak *et al*., 2000). All of these *Prevotella* strains are reported to show high genetic diversity on the basis of their 16S rRNA sequences (Avguštin *et al*., 1994; 1997; Ramšak *et al*., 2000; Bekele *et al*., 2010), although it is possible that these organisms show extensive functional redundancy and perform the same role in the rumen. In the absence of genome sequence information or experimental evidence this remains unproven. In addition to the family *Prevotellaceae*, Figs 1 and 3 also show that many other members of the *Bacteroidetes* are found in the rumen. These have largely escaped isolation and represent the largest group according to our study which is missing cultured representatives. Five clades (1, 3, 4, 5, and 6 in Fig. 3) together make up 6% of the bacterial population in our study but have no cultured representatives. Many of these have a closest, albeit weak, match to organisms belonging to the families *Porphyromonadaceae* and *Rikenellaceae* which are often asaccharolytic and so may elude culture in studies that focus on plant polysaccharide degrading fractions of the rumen. There is a need for a renewed cultivation effort into these taxa.

### Bacterial abundance and novelty

To examine the most abundant and most diverse (distinct from available cultures) OTUs from this study, we plotted the phylogenetic distance against the average abundance for each OTU and coloured according to the prevalence (Fig. 2A). A total of 27 OTUs had a scaled abundance of greater than 0.25 and are believed to represent the most numerous OTUs from the rumen datasets analysed (Supporting Information Table S5A). Eight of these OTUs were also part of the core microbiome described in the next section. The 27 OTUs belong to three bacterial phyla, *Bacteroidetes* 13 OTUs (48%), *Firmicutes* 11 OTUs (41%) and *Proteobacteria* 3 OTUs (11%). Of the 13 abundant OTUs assigned to the phyla *Bacteroidetes* (Supporting Information Fig. S1), nine were classified to the family *Prevotellaceae*, although they do not have close homology to the four *Prevotella* species described from the rumen. The remaining four OTUs can only be classified as uncultured bacteria within the order *Bacteroidales*. The most abundant OTU from the phylum *Firmicutes* is OTU121 belonging to the genus *Succiniclasticum* (family *Acidaminococcaceae*) and displaying 96% gene sequence similarity with the 16S rRNA gene from the type strain of *Succiniclasticum ruminis*. *S. ruminis* is reported to be a common inhabitant of the rumen and is characterized by the ability to ferment succinate to propionate without a requirement for other carbohydrates (Van Gylswyk, 1995). Other abundant *Firmicutes* OTUs are OTU561 identified as *Kandleria vitulina* (Salvetti *et al*., 2011), OTU1618, OTU1892 and OTU1556, all with sequences similar to that deposited for rumen bacterium R-7 (Supporting Information Fig. S2). These bacteria potentially represent a new family in the order *Clostridiales*. The closest associations are to *Catabacteriaceae* and *Christensenellaceae*, but further analysis is needed to determine the true placement of this group. OTU2179 shows 100% gene sequence similarity with the 16S rRNA gene from the rumen bacterium R-25. Bacteria similar to R-25 have been shown to be prevalent in the rumen (Koike *et al*., 2010) and contribute to hemicellulose breakdown. Co-culture of strain R-25 with *Fibrobacter succinogenes* showed synergism between the two bacteria resulting in increased digestion of rice straw (Fukuma *et al*., 2012). Two other *Firmicutes* OTUs occur only in single datasets and belong to the lactic acid bacteria. These are OTU807 (*Lactobacillus helveticus*) and OTU609 (*Carnobacterium* sp.). It is not known if these are common rumen inhabitants or whether they are transient organisms introduced with the feed. Other abundant OTUs belong to unnamed genera within the families *Lachnospiraceae* and *Ruminococcaceae*. The final phylum in terms of abundance is *Proteobacteria*. The single most abundant OTU (OTU8246) could only be identified to the family level (*Succinovibrionaceae*), but closely matching clone sequences have been reported in a study of bacteria from the bovine rumen epithelium (Li *et al*., 2012). The other two *Proteobacteria* OTUs both match to the genus *Psychrobacter*, although there are no cultured isolates belonging to this genus from the rumen. Sequences matching to OTU8536 represent an unnamed *Psychrobacter* species and are also found in a study of rice chaff composted with dairy manure (Tian *et al*., 2013) while OTU8698 matches to *Psychrobacter faecalis* at 99% identity.

To identify novelty in the rumen datasets analysed we considered the most diverse OTUs to have a scaled phylogenetic distance greater than 0.25 (Supporting Information Table S5B). Similar to abundance, the majority appear to fall within the phyla *Firmicutes* (37%), *Bacteriodetes* (35%) and *Proteobacteria* (12%), although we do see the appearance of other phyla in this category, such as *Spirochaetes*, *Chloroflexi*, *Tenericutes* and *Acidobacteria*. As expected, almost all the identified OTUs do not have cultured rumen isolates (Supporting Information Figs S1 and S2) and will require further cultivation efforts to establish their contribution to rumen function. Based on our study, most of this group are not prevalent in the rumen microbiome with the exception of two identified OTUs which are discussed in the core microbiome section later. Targeting these types of organisms will lead to a greater understanding of the diversity of rumen microbes, but the contribution these organisms make to rumen function awaits further investigation.

### Rumen bacterial core microbiome

In this study, we identified 98 OTUs which we consider are part of the rumen bacterial core microbiome based on their prevalence in at least five of the seven datasets studied and therefore likely represent those fundamental to the function of the rumen (Fig. 2B; Supporting Information Table S6). The number of core OTUs discovered from each of the studies was found to be related to the initial number of sequences, rather than the region targeted and no bias for shorter targeted regions was found (Supporting Information Fig. S3). These observations predict that approximately 200 000 initial reads are required to guarantee observation of 90% of the core OTUs (Supporting Information Fig. S3). The 98 OTUs can be classified into two bacterial phyla, *Firmicutes*, 57 OTUs (58%), and *Bacteroidetes* 41 OTUs (42%). Of the 57 *Firmicutes* OTUs (Supporting Information Fig. S2), 18 OTUs had a 16S rRNA gene sequence that shared > 96% gene sequence similarity with 16S rRNA genes from bacteria belonging to five families, *Lachnospiraceae*, *Ruminococcaceae*, *Streptococcaceae*, *Veillonellaceae* and *Acidaminococcaceae*. Eleven of these OTUs could be further classified to the genus level. They included members of the genera *Butyrivibrio*, *Lachnobacterium*, *Oribacterium*, *Ruminococcus*, *Selenomonas*, *Streptococcus* and *Succiniclasticum*. Seven OTUs show similarity to unnamed and potentially novel genera described in the papers by Koike and colleagues (2010), Kenters and colleagues (2011) and Nyonyo and colleagues (2013). Thirteen OTUs had a 16S rRNA gene sequence that shared between 93–96% gene sequence similarity with 16S rRNA genes from two bacterial families, *Lachnospiraceae* and *Ruminococcaceae*. An additional two OTUs (OTU1618 and OTU1888) could not be assigned to a named bacterial family. These OTUs display similarity to the sequence deposited for rumen bacterium R-7. The remaining 24 OTUs are considered to have no close relatives as they have 16S rRNA gene sequence similarities of between 81–92%, which suggest they belong to genera that have not yet been adequately described. Based on family classifications, most belong to *Lachnospiraceae* and *Ruminococcaceae* while others show closest homology to the families *Erysipelotrichaeceae*, *Oscillospiraceae*, *Clostridiales* Family_XIII *incertae sedis* and the R-7-like organisms described earlier.

Forty-one OTUs are classified to the phylum *Bacteriodetes* (Supporting Information Fig. S1). Of these, six OTUs had a 16S rRNA gene sequence that shared > 96% gene sequence similarity with 16S rRNA genes from one bacterial family, *Prevotellaceae*. A further 19 OTUs had 16S rRNA gene sequences that shared between 93–96% similarity with members of the family *Prevotellaceae*. The remaining 16 OTUs showed no clear similarity to relatives based on 16S rRNA gene sequence similarities of between 87–92%. The closest homology is to members of the families *Prevotellaceae, Porphyromonadaceae* and *Rikenellaceae.*

To examine abundance in the identified core rumen bacterial microbiota we investigated the top 10 OTUs based on their ranked abundance and revealed three OTUs classified to the phylum *Firmicutes* and seven OTUs to the phylum *Bacteriodetes*. The most abundant OTU (OTU121) is identified as being from the genus *Succiniclasticum*, displaying 96% gene sequence similarity with the 16S rRNA gene from the type strain *Succiniclasticum ruminis*. Other *Firmicutes* OTUs in the top 10 most abundant are OTU1618 predicted to be a rumen bacterium R-7-like organism and OTU2179 which shows 100% gene sequence similarity with the 16S rRNA gene from the rumen bacterium R-25. The remaining top 10 most abundant OTUs all classify to the family *Prevotellaceae* but do not have close homology to the four species described for the rumen.

Many of the best studied bacteria, often isolated because of their fibre-degrading ability, are not among the most abundant. For example, the cellulolytic *Ruminococcus flavefaciens* was identified among the core microbiota bacteria but only occurs at low numbers (Fig. 2 and Supporting Information Table S6). Consequently *R. flavefaciens*, and possibly the other cellulolytic bacteria, may be regarded as ‘keystone’ species whose numbers do not reflect their importance to the rumen environment (Ze *et al*., 2013).

To analyse for novelty we explored the top 10 OTUs based on their ranked phylogenetic distance to the nearest RDP isolate and identified six OTUs classified to the phylum *Firmicutes* and four OTUs to the phylum *Bacteriodetes*. OTU3893 is considered the most novel in our core microbiota but still falls within the family *Prevotellaceae*. Three other OTUs classified to the phylum *Bacteriodetes* are also in this top 10. They show closest homology to the family *Porphyromonadaceae*. Six OTUs from the phylum *Firmicutes* round up the top 10. Four are considered members of the family *Lachnospiraceae* while the other two belong to the *Erysipelotrichaeceae* and *Ruminococcaceae*. Examination of the 98 core OTUs in terms of presence in datasets sampled shows two OTUs present in seven datasets, 27 OTUs present in six datasets and 69 OTUs present in five datasets. The two OTUs found in all seven datasets are OTU2004 and OTU2133 (Supporting Information Fig. S2). Both OTUs represent novel genera within the family *Ruminococcaceae* as previously described by Kenters and colleagues (2011). The nearest cultured isolate for OTU2004 is rumen bacterium NK3A31 at 96% percent identity and the nearest cultured isolate for OTU2133 is rumen bacterium NK4A214 at 95% percent identity.

### Cultured rumen bacteria of interest

The goals of this study were to identify cultured rumen bacterial taxa that have yet to be the subject of genome sequencing studies, and to identify taxa that require more cultivation effort. Based on our collated information for cultured rumen bacteria, it is shown that many genera still remain without genome sequence information. We have compiled a list of cultured rumen isolates available in public culture collections or reported to be in culture (Table 2). This includes 42 candidate rumen bacterial cultures potentially available for genome sequencing. Additionally, we have used 16S rRNA-based studies to identify 157 abundant, novel and core OTUs from the rumen microbiota. Detailed phylogenetic trees of the core, abundant and novel OTUs and related cultured representative sequences from the phyla *Firmicutes* and *Bacteriodetes* are shown in Supporting Information Figs S1 and S2. OTUs were widely distributed across the trees, often tended to form deep branches, and did not always form coherent clusters with cultured relatives. Sixty-five OTUs had > 93% gene sequence similarity with 16S rRNA genes from cultured rumen bacteria and were used to select for additional cultures for genome sequencing. With the removal of those selected cultured rumen bacteria where genome sequencing is already targeted or in progress and the removal of duplication where an OTU showed top homology to the same rumen cultured isolate, this resulted in an additional 24 candidate rumen cultures being added to the list (Table 2). The remaining 92 abundant, novel and core OTUs from the rumen bacterial microbiota do not show high homology to cultured rumen organisms and as such represent taxa that require further cultivation effort. At this stage, we can only confidently assign these OTUs to the phylum level. The majority falls within the *Firmicutes*, *Bacteriodetes* and *Proteobacteria* reflecting the overall composition of the rumen bacterial microbiota. These organisms are not represented among culture collections suggesting that more intensive culturing efforts incorporating new culture and single-cell-based approaches will be required to isolate these organisms for sequencing. It is clear from our study that rumen microbes from the Bacteriodetes phylum are under-represented in culture. A further four candidate rumen isolates from this phylum are included in Table 2 which are outside the most abundant clades shown in Fig. 3. It is hoped that genome sequencing of organisms such as these will help elucidate the overall functional diversity of rumen Bacteriodetes.

**Table 2:** List of cultured rumen bacteria of interest

Phylum	Family	Candidate cultures
*Actinobacteria*	*Actinomycetaceae*	*Actinomyces ruminicola* B71
*Actinobacteria*	*Coriobacteriaceae*	*Atopobium* sp. G44
*Actinobacteria*	*Cellulomonadaceae*	*Cellulomonas* sp. N-14
*Actinobacteria*	*Coriobacteriaceae*	*Denitrobacterium detoxificans* NP0H1T
*Actinobacteria*	*Micromonosporaceae*	*Micromonospora ruminantium* C1
*Actinobacteria*	*Coriobacteriaceae*	*Olsenella umbonata* A2
*Actinobacteria*	*Pseudonocardiaceae*	*Prauserella rugosa* IMRU 3760T
*Bacteriodetes*	Unclassified *Bacteriodetes*	*Ruminobacillus xylanolyticum* G1
*Bacteriodetes*	*Rikenellaceae*	*Ruminofilibacter xylanolyticum* S1
*Bacteroidetes*	*Sphingobacteriaceae*	*Shingobacterium thalpophilum* Y4
*Bacteroidetes*	*Prevotellaceae*	*Prevotella ruminicola* Tc2-24
*Bacteroidetes*	*Prevotellaceae*	*Prevotella ruminicola* BP1-34
*Bacteroidetes*	*Prevotellaceae*	*Prevotella ruminicola* BP5-11
*Bacteroidetes*	*Prevotellaceae*	*Prevotella ruminicola* TC2-28
*Bacteroidetes*	*Prevotellaceae*	*Prevotella ruminicola* TF2-5
*Bacteroidetes*	*Prevotellaceae*	*Prevotella ruminicola* BP1-162
*Bacteroidetes*	*Prevotellaceae*	*Prevotella* sp. BP1-145
*Bacteroidetes*	*Prevotellaceae*	*Prevotella* sp. BP1-148
*Bacteroidetes*	*Prevotellaceae*	*Prevotella* sp. BP1-56
*Bacteroidetes*	*Prevotellaceae*	*Prevotella* sp. BP1-57
*Bacteroidetes*	*Prevotellaceae*	*Prevotella* sp. R79
*Bacteroidetes*	*Prevotellaceae*	*Prevotella* sp. RM13
*Bacteroidetes*	*Prevotellaceae*	*Prevotella* sp. RM47
*Bacteroidetes*	*Prevotellaceae*	rumen bacterium NK4A111
*Bacteroidetes*	*Prevotellaceae*	rumen bacterium R-9
*Bacteroidetes*	*Prevotellaceae*	*Prevotella ruminicola* 223/M2/7
*Bacteroidetes*	*Prevotellaceae*	rumen bacterium NK4A62
*Bacteroidetes*	Unclassified *Bacteriodetes*	Bacteroidales bacterium RM68
*Bacteroidetes*	Unclassified *Bacteriodetes*	Bacteroidales bacterium RM69
*Bacteroidetes*	Unclassified *Bacteriodetes*	Bacteroidales bacterium RM8
*Bacteroidetes*	Unclassified *Bacteriodetes*	Bacteroidales bacterium R61
*Firmicutes*	*Acidaminococcaceae*	*Acidaminococcus fermentans* H18
*Firmicutes*	*Veillonellaceae*	*Allisonella histaminiformans* MR2T
*Firmicutes*	*Clostridiales* Family XIII. *Incertae Sedis*	*Anaerovorax* sp. G61
*Firmicutes*	*Lachnospiraceae*	*Cellulosilyticum ruminicola* H1T
*Firmicutes*	*Lachnospiraceae*	*Coprococcus* sp. PE15
*Firmicutes*	Unclassified *Clostridiales*	*Howardella ureilytica* GPC589T
*Firmicutes*	*Leuconostocaceae*	*Leuconostoc mesenteroides* L.JST
*Firmicutes*	*Clostridiaceae*	*Oxobacter pfennigii* V5-2T
*Firmicutes*	*Paenibacillaceae*	*Paenibacillus ruminocola* CA8
*Firmicutes*	*Clostridiales* Family XI. *Incertae Sedis*	*Peptoniphilus* sp. BG5
*Firmicutes*	*Peptostreptococcaceae*	*Peptostreptococcus anaerobius*
*Firmicutes*	*Clostridiaceae*	*Proteiniclasticum ruminis* D3RC-2
*Firmicutes*	*Veillonellaceae*	*Quinella ovalis*
*Firmicutes*	*Clostridiaceae*	*Saccharofermentans* sp. G8
*Firmicutes*	*Clostridiaceae*	*Sarcina* sp. LD64
*Firmicutes*	*Lachnospiraceae*	*Syntrophococcus sucromutans* S195T
*Firmicutes*	*Clostridiales* Family XI. *Incertae Sedis*	*Tissierella* sp. S2
*Firmicutes*	*Veillonellaceae*	*Veillonella* sp. FMH104
*Firmicutes*	*Lachnospiraceae*	*Clostridium aminophilum* 152R-1b
*Firmicutes*	*Lachnospiraceae*	*Eubacterium cellulosolvens* Ce2
*Firmicutes*	*Lachnospiraceae*	*Lachnospiraceae* bacterium RM66
*Firmicutes*	*Ruminococcaceae*	rumen bacterium NK3A31
*Firmicutes*	*Ruminococcaceae*	rumen bacterium NK4A214
*Firmicutes*	Unclassified *Clostridiales*	rumen bacterium R-7
*Proteobacteria*	*Enterobacteriaceae*	*Proteus vulgaris* BR_22
*Proteobacteria*	*Moraxellaceae*	*Acinetobacter* sp. IVS-W
*Proteobacteria*	*Neisseriaceae*	*Alysiella filiformis* A1T
*Proteobacteria*	*Xanthobacteraceae*	*Ancylobacter* sp. ECPB09
*Proteobacteria*	*Campylobacteraceae*	*Campylobacter* sp. FF05
*Proteobacteria*	*Enterobacteriaceae*	*Escherichia* sp. FHM113
*Proteobacteria*	*Enterobacteriaceae*	*Klebsiella* sp. KG1
*Proteobacteria*	*Pasteurellaceae*	*Mannheimia ruminalis* HPA92T
*Proteobacteria*	*Oxalobacteraceae*	*Oxalobacter formigenes* OxB
*Proteobacteria*	*Alcaligenaceae*	*Pigmentiphaga* sp. ECPB08
*Proteobacteria*	*Pseudomonadaceae*	*Pseudomonas putida* BR_1
*Proteobacteria*	*Enterobacteriaceae*	*Shigella flexneri* G3
*Proteobacteria*	*Moraxellaceae*	bacterium XJ141-10-NGI
*Tenericutes*	*Anaeroplasmataceae*	*Anaeroplasma abactoclasticum*
*Tenericutes*	*Anaeroplasmataceae*	*Asteroleplasma anaerobium*

## Conclusions

Taking a collective approach of utilizing information gained from culture collections, scientific literature, and the NCBI and RDP databases, and linking this with a comparative study of several rumen 16S rRNA gene-based surveys, we have attempted to capture a snapshot of rumen bacterial diversity. We are aware that this study may not reflect the full diversity of rumen microbes and will be biased to the datasets sampled; however, it provides a reference point for the Hungate1000 which will be further developed when information from more comprehensive studies, such as the Global Rumen Census (globalrumencensus.org.nz), becomes available.

Genome sequence information is available for a limited number of rumen bacteria (Leahy *et al*., 2013, Supporting Information Table S1), with particular emphasis on those organisms involved in the breakdown of plant cell wall polysaccharides. While these sequenced bacteria include members of 20 different bacterial genera (Supporting Information Table S1) they only begin to address the taxonomic and functional diversity present in the rumen. Many of the remaining rumen bacteria known to be in culture are now, or will be, targeted by community-sequencing programmes such as the Hungate1000 and GEBA.

Community profiling based on analysis of 16S rRNA genes will continue to address the questions of ‘Who is there?’ and ‘How many’, and metagenomic/metatranscriptomic analyses make it possible to catalogue the genes present and determine which ones are expressed. However, genome sequences can reveal much about a microbe’s metabolic potential and go a long way to addressing the question of ‘What are they doing?’ If genomic studies are to make an impact on our understanding of rumen microbial ecology and how specific rumen microbes interact with the animal, the feed, or each other, there needs to be an additional focus on bringing organisms into cultivation with subsequent detailed study and characterization. Isolation of strains that represent OTUs that are both abundant and novel or abundant and likely to belong to the core microbiome should be prioritized. Cultivation and isolation of such strains remains the biggest challenge, and a firm grounding in basic principles, such as bacterial physiology and metabolism, is required to exploit the data generated. It is more than 50 years ago that Marvin Bryant concluded that most of the more significant groups (of rumen bacteria) had been isolated (Bryant, 1959), and while most of our current knowledge derives from the cultures isolated in these pioneering studies, it is time to re-examine the diversity of bacteria present using the tools that are now available. This paper will hopefully stimulate enthusiasm for the isolation and study of rumen bacteria in culture and allow the Hungate1000 to target a true representation of the diversity and novelty of the rumen bacterial microbiome.

## Experimental procedures

### Sequenced data retrieval, filtering and clustering

The sequences deposited from nine independent large-scale 16S studies from the rumen (Brulc *et al*., 2009; Kong *et al*., 2010; Pitta *et al*., 2010; De Menezes *et al*., 2011; Hess *et al*., 2011; Li *et al*., 2011; Jami and Mizrahi, 2012; Kav *et al*., 2012; Pope *et al*., 2012) were retrieved. The regions of the 16S rRNA genes sequenced were determined for each dataset and simultaneously screened for possible reverse complementarity using V-REVCOMP (Hartmann *et al*., 2011). Those seven datasets that were found to overlap for the V1 and V2 regions at a minimum were retained (Supporting Information Table S3); the other two datasets (Hess *et al*., 2011; Li *et al*., 2011) were not included in any further analyses. Each of the seven datasets were individually processed as follows: The raw sequences were clustered at 97% identity using the CD-HIT (Fu *et al*., 2012) package. The OTUs identified were then profile-aligned using the ‘align_to_rRNA_profile.pl’ script from the software package stap (Wu *et al*., 2008) and the alignment visualized using Jalview (Waterhouse *et al*., 2009). Both 5′ and 3′ conserved regions across all the OTUs were identified and these were used to trim the raw sequences to homologous sequence regions using in-house scripts. Any sequences that did not contain both conserved regions were excluded and any under dataset-specific minimum sequence lengths were also removed. The trimmed and filtered raw reads were then clustered at 97% identity using either CD-HIT or CD-HIT-OTU (Fu *et al*., 2012) depending on the dataset (see Supporting Information Table S3 for details). These are referred to as the *de novo* datasets in the following sections.

### Cultured sequence data retrieval, filtering and clustering

All bacterial sequences that met the following criteria were retrieved from the RDP database (Cole *et al*., 2014). Strain: Both Type and Non-type, Source: Isolates, Size: greater or equal to 1200 bp, Quality: Good. All sequences were clustered at 97% identity using CD-HIT and a single representative for each cluster was used to represent the diversity of isolated bacteria and used for further analyses.

### Identifying universal OTUs

The OTUs from each of the seven datasets were then combined with the OTUs from the RDP sequences and profile-aligned using stap and visualized using Jalview. All sequences were trimmed to the same length and clustered at 97% using CD-HIT to identify universal OTUs. The sequences from the *de novo* datasets and RDP representatives were combined for any OTUs that were clustered together. The advantage of this two-tiered approach is that dataset-specific clustering could be carried out using all the 16S rRNA regions available for each rather then clustering all the sequences together using the maximum regions that overlapped across all seven datasets. Chimeric reads were detected using cd-hit-dup from the CD-HIT package, which recognizes parent reads of a chimera through the similarity of their cross-over to a potential chimera (and where the original parent reads are sufficiently dissimilar to the chimera).

### Phylogenetic analysis

The universal OTUs were all profile-aligned using stap and visualized using Jalview. The alignment was manually checked and adjusted for regions that were obviously mis-aligned and sites containing gaps for the majority of OTUs were removed. The trimmed alignment was used as input to FastTree (Price *et al*., 2010) to estimate a phylogeny. Tip-to-tip distances across the tree were calculated between all taxa using an in-house script, and for each OTU from clusters containing sequences from a *de novo* dataset, the shortest distance to an OTU from a cluster containing an RDP sequence (and therefore a cultured isolate) was recorded. This value was set to zero if the OTU came from a cluster that contained an RDP sequence.

To produce a tree that only contained the OTUs from the *de novo* datasets, all other OTUs were pruned from the tree while preserving the branch lengths, using Clann (Creevey and McInerney, 2005); this tree was visualized using iTOL (Letunic and Bork, 2011) and is shown in Fig. 1. A second phylogeny of only the *Bacteriodetes*, containing the OTUs from both the *de novo* datasets and the RDP database was similarly constructed by pruning the relevant taxa from the large 22 031 taxon tree and visualized using iTOL. Taxonomic assignments for all the OTUs was carried out using the RDP classifier (Cole *et al*., 2014).

Sequences of cultures related to *Bacteroidetes* and *Firmicutes* 16S rRNA novel, abundant (Supporting Information Table S5), or core (Supporting Information Table S6) OTUs were retrieved from the SILVA SSURef database (version 111; Quast *et al*., 2013) and GenBank (Benson *et al*., 2007) and aligned against the SILVA database (SINA version 1.2.11; Pruesse *et al*., 2012). The alignment was manually refined in arb (version 5.3; Ludwig *et al*., 2004). Phylogenetic trees of *Bacteroidetes* or *Firmicutes* sequences were inferred using maximum likelihood in combination with the GTRGAMMA nucleotide substitution model and rapid bootstrap analysis (RAxML version 7.3.2; Stamatakis, 2006). *Butyrivibrio proteoclasticus* strain B316^T^ (CP001810) and *Prevotella ruminicola* strain Bryant 23^T^ (L16482) were used as outgroups respectively. Bootstrap values were calculated from 1000 replicates. Novel, abundant and core OTUs were mapped onto these trees using the parsimony insertion tool in arb.

### Summary statistics

Prevalence of each universal OTU was calculated as the proportion of *de novo* datasets from which the dataset was identified. Average abundance of each OTU was calculated by first scaling the number of sequences from each dataset using the trimmed mean of M (TMM) method and then calculating the average percentage abundance of each across all the datasets. To produce the graphs in Fig. 2, the phylogenetic distances to the nearest RDP sequence for each OTU was reported as the ratio to the largest phylogenetic distance calculated for the entire dataset. Similarly the scaled abundance for each OTU was reported as the ratio to the largest OTU in the dataset. This allowed a scaled ranking of OTUs which could be plotted on both axes within the range 0 to 1. Euclidean distances were then calculated for each OTU as the distance from the (0,0) point on the graph using these values. The results of these calculations are outlined in supplementary Supporting Information Table S7.

## Supporting Information

Figure S1 Phylogenetic placement of Bacteroidetes-affiliated novel [N], abundant [(A) Supporting Information Table S5], and core [(C) Supporting Information Table S6] rumen operational taxonomic units (OTUs) in relation to 16S rRNA gene reference sequences of related cultured strains. The phylogenetic tree was inferred from reference sequences using the maximum-likelihood method in combination with a GTRGAMMA substitution model implemented in RAxML. *Butyrivibrio proteoclasticus strain* B316T (CP001810) was used as an outgroup. Bootstrap values were calculated from 1000 replicates; only values greater than 70% are shown. The scale bar corresponds to the mean number of nucleotide substitutions per site. Novel, abundant and core OTUs were mapped onto these trees using the parsimony insertion tool in arb. [T] indicates a type strain.

Figure S2 Phylogenetic placement of *Firmicutes*-affiliated novel [N], abundant [(A) Supporting Information Table S5], and core [(C) Supporting Information Table S6] rumen operational taxonomic units (OTUs) in relation to 16S rRNA gene reference sequences of related cultured strains. The phylogenetic tree was inferred from reference sequences using the maximum-likelihood method in combination with a GTRGAMMA substitution model implemented in RAxML. *Prevotella ruminicola* strain Bryant 23T (L16482) was used as an outgroup. Bootstrap values were calculated from 1000 replicates; only values greater than 70% are shown. The scale bar corresponds to the mean number of nucleotide substitutions per site. Novel, abundant and core OTUs were mapped onto these trees using the parsimony insertion tool in arb. [T] indicates a type strain.

Figure S3 Scatter plot of the number of core OTUs found from each rumen dataset examined, compared with the number of initial reads sequenced. The labels on the points illustrate from which study each was derived and the region of the 16S targeted (see Supporting Information Table S3 for more details).

**Table S1.** List of genera with reported cultured rumen isolates. **Table S2.** List of cultured rumen isolates identified in culture collections surveyed. Culture collections were searched in this order DSMZ- >ATCC- >JCM- >BCCM/LMG- >CCUG. Several cultures are desposited in more than one collection, the first reference number is listed. **Table S3.** Data from de novo studies used in this analysis. **Table S4.** Summary statistics of the 300 monophyletic clades or singletons identified in this study. **Table S5.** List of most abundant and most diverse OTUs identified in this study. **Table S6.** List of the core rumen bacterial OTUs identified in this study. **Table S7.** Summary statistics of the OTUs identified in this study.

## References

[b1] Avguštin G, Wright F, Flint HJ (1994). Genetic diversity and phylogenetic relationships among strains of *Prevotella**Bacteroides**ruminicola* from the rumen. Int J Syst Bacteriol.

[b2] Avguštin G, Wallace RJ, Flint HJ (1997). Phenotypic diversity among ruminal isolates of *Prevotella ruminicola*: proposal of *Prevotella brevis* sp. *nov**Prevotella bryantii* sp. *nov*., and *Prevotella albensis* sp. *nov*. and redefinition of *Prevotella ruminicola*. Int J Syst Bacteriol.

[b3] Bekele AZ, Koike S, Kobayashi Y (2010). Genetic diversity and diet specificity of ruminal *Prevotella* revealed by 16S rRNA gene-based analysis. FEMS Microbiol Lett.

[b4] Benson DA, Karsch-Mizrachi I, Lipman DJ, Ostell J, Wheeler DL (2007). GenBank. Nucleic Acids Res.

[b5] Brulc JM, Antonopoulos DA, Miller ME, Wilson MK, Yannarell AC, Dinsdale EA (2009). Gene-centric metagenomics of the fiber-adherent bovine rumen microbiome reveals forage specific glycoside hydrolases. Proc Natl Acad Sci USA.

[b6] Bryant MP (1959). Bacterial species of the rumen. Bacteriol Rev.

[b7] Clarke RTJ (1979). Niche in pasture-fed ruminants for the large rumen bacteria *Oscillospira**Lampropedia*, and Quin’s and Eadie’s ovals. Appl Environ Microbiol.

[b8] Cole JR, Wang Q, Fish JA, Chai B, McGarrell DM, Sun Y (2014). Ribosomal database project: data and tools for high throughput rRNA analysis. Nucleic Acids Res.

[b9] Creevey CJ, McInerney JO (2005). Clann: investigating phylogenetic information through supertree analyses. Bioinformatics.

[b10] De Menezes AB, Lewis E, O’Donovan M, O’Neill BF, Clipson N, Doyle EM (2011). Microbiome analysis of dairy cows fed pasture or total mixed ration diets. FEMS Microbiol Ecol.

[b11] Fouts DE, Szpakowski S, Purushe J, Torralba M, Waterman RC, MacNeil MD (2012). Next generation sequencing to define prokaryotic and fungal diversity in the bovine rumen. PLoS ONE.

[b12] Fu L, Niu B, Zhu Z, Wu S, Li W (2012). CD-HIT: accelerated for clustering the next-generation sequencing data. Bioinformatics.

[b13] Fukuma N, Koike S, Kobayashi Y (2012). Involvement of recently cultured group U2 bacterium in ruminal fiber digestion revealed by coculture with *Fibrobacter succinogenes* S85. FEMS Microbiol Lett.

[b14] Hartmann M, Howes CG, Veldre V, Schneider S, Vaishampayan PA, Yannarell AC (2011). V-REVCOMP: automated high-throughput detection of reverse complementary16S rRNA gene sequences in large environmental and taxonomic datasets. FEMS Microbiol Lett.

[b15] Hess M, Sczyrba A, Egan R, Kim TW, Chokhawala H, Schroth G (2011). Metagenomic discovery of biomass-degrading genes and genomes from cow rumen. Science.

[b16] Hungate RE (1966). The Rumen and Its Microbes.

[b17] Jami E, Mizrahi I (2012). Composition and similarity of bovine rumen microbiota across individual animals. PLoS ONE.

[b18] Kav A, Sasson G, Brown A, Jami E, Doron-Faigenboim A, Benhar I, Mizrahi I (2012). Insights into the bovine rumen plasmidome. Proc Natl Acad Sci USA.

[b19] Kenters N, Henderson G, Jeyanathan J, Kittelmann S, Janssen PH (2011). Isolation of previously uncultured rumen bacteria by dilution to extinction using a new liquid culture medium. J Microbiol Methods.

[b20] Kim M, Morrison M, Yu Z (2011). Status of the phylogenetic diversity census of ruminal microbiomes. FEMS Microbiol Ecol.

[b21] Kingston-Smith AH, Davies TE, Stevens PR, Mur LAJ (2013). Comparative metabolite fingerprinting of the rumen system during colonization of three forage grass (*Lolium perenne* L.) varieties. PLoS ONE.

[b22] Koike S, Handa Y, Goto H, Sakai K, Miyagawa E, Matsui H (2010). Molecular monitoring and isolation of previously uncultured bacterial strains from the sheep rumen. Appl Environ Microbiol.

[b23] Kong Y, Teather R, Forster R (2010). Composition, spatial distribution, and diversity of the bacterial communities in the rumen of cows fed different forages. FEMS Microbiol Ecol.

[b24] Leahy SC, Kelly WJ, Ronimus RS, Wedlock N, Altermann E, Attwood GT (2013). Genome sequencing of rumen bacteria and archaea and its application to methane mitigation strategies. Animal.

[b25] Letunic I, Bork P (2011). Interactive Tree Of Life v2: online annotation and display of phylogenetic trees made easy. Nucleic Acids Res.

[b26] Li M, Zhou M, Adamowicz E, Basarab JA, Guan LL (2012). Characterization of bovine ruminal epithelial bacterial communities using 16S rRNA sequencing, PCR-DGGE, and qRT-PCR analysis. Vet Microbiol.

[b27] Li RW, Connor EE, Li C, Baldwin RL, Sparks ME (2011). Characterization of the rumen microbiota of pre-ruminant calves using metagenomic tools. Environ Microbiol.

[b28] Ludwig W, Strunk O, Westram R, Richter L, Meier H, Yadhukumar (2004). arb: a software environment for sequence data. Nucleic Acids Res.

[b29] Noel S (2013).

[b30] Nyonyo T, Shinkai T, Tajima A, Mitsumori M (2013). Effect of media composition, including gelling agents, on isolation of previously uncultured rumen bacteria. Lett Appl Microbiol.

[b31] Pitta DW, Pinchak E, Dowd SE, Osterstock J, Gontcharova V, Youn E (2010). Rumen bacterial diversity dynamics associated with changing from bermuda grass hay to grazed winter wheat diets. Microb Ecol.

[b32] Pope PB, Mackenzie AK, Gregor I, Smith W, Sundset MA, McHardy AC (2012). Metagenomics of the Svalbard reindeer rumen microbiome reveals abundance of polysaccharide utilization loci. PLoS ONE.

[b33] Price MN, Dehal PS, Arkin AP (2010). FastTree 2 – approximately maximum-likelihood trees for large alignments. PLoS ONE.

[b34] Pruesse E, Peplies J, Glockner FO (2012). SINA: accurate high-throughput multiple sequence alignment of ribosomal RNA genes. Bioinformatics.

[b35] Quast C, Pruesse E, Yilmaz P, Gerken J, Schweer T, Yarza P (2013). The SILVA ribosomal RNA gene database project: improved data processing and web-based tools. Nucleic Acids Res.

[b36] Ramšak A, Peterka M, Tajima K, Martin JC, Wood J, Johnston ME (2000). Unravelling the genetic diversity of ruminal bacteria belonging to the CFB phylum. FEMS Microbiol Ecol.

[b37] Salvetti E, Felis GE, Dellaglio F, Castioni A, Torriani S, Lawson PA (2011). Reclassification of *Lactobacillus catenaformis* (Eggerth 1935) Moore and Holdeman 1970 and *Lactobacillus vitulinus* Sharpe *et al*. 1973 as *Eggerthia catenaformis* gen. nov., comb. nov. and *Kandleria vitulina* gen. nov., comb. nov., respectively. Int J Syst Evol Microbiol.

[b38] Stamatakis A (2006). RAxML-VI-HPC: maximum likelihood-based phylogenetic analyses with thousands of taxa and mixed models. Bioinformatics.

[b39] Stevenson DM, Weimer PJ (2007). Dominance of *Prevotella* and low abundance of classical ruminal bacterial species in the bovine rumen revealed by relative quantification real-time PCR. Appl Microbiol Biotechnol.

[b40] Tian W, Sun Q, Xu D, Zhang Z, Chen D, Li C (2013). Succession of bacterial communities during composting process as detected by 16S rRNA clone libraries analysis. Int Biodeterior Biodegradation.

[b41] Van Gylswyk NO (1995). *Succiniclasticum ruminis* gen. *nov.,* sp*. nov*., a ruminal bacterium converting succinate to propionate as the sole energy-yielding mechanism. Int J Syst Bacteriol.

[b42] Waterhouse AM, Procter JB, Martin DMA, Clamp M, Barton GJ (2009). Jalview Version 2 – a multiple sequence alignment editor and analysis workbench. Bioinformatics.

[b43] Wu D, Hartman A, Ward N, Eisen JA (2008). An automated phylogenetic tree-based small subunit rRNA taxonomy and alignment pipeline (STAP). PLoS ONE.

[b44] Wu D, Hugenholtz P, Mavromatis K, Pukall R, Dalin E, Ivanova NN (2009). A phylogeny-driven genomic encyclopaedia of Bacteria and Archaea. Nature.

[b45] Wu S, Baldwin RL, Li W, Li C, Connor EE, Li RW (2012). The bacterial community composition of the bovine rumen detected using pyrosequencing of 16S rRNA genes. Metagenomics.

[b46] Yutin N, Galperin MY (2013). A genomic update on clostridial phylogeny: gram-negative spore formers and other misplaced clostridia. Environ Microbiol.

[b47] Ze X, Le Mougen F, Duncan SH, Louis P, Flint HJ (2013). The role of ‘keystone’ species in the degradation of recalcitrant substrates. Gut Microbes.

[b48] Ziemer CJ (2014). Newly cultured bacteria with broad diversity isolated from 8 week continuous culture enrichments of cow feces on complex polysaccharides. Appl Environ Microbiol.

