# Supplementary figures and images for: Determining the culturability of the rumen bacterial microbiome
Source: Microb Biotechnol. 2014 Jul 1;7(5):467–79. doi: 10.1111/1751-7915.12141 (PMC4229327; doi:10.1111/1751-7915.12141)

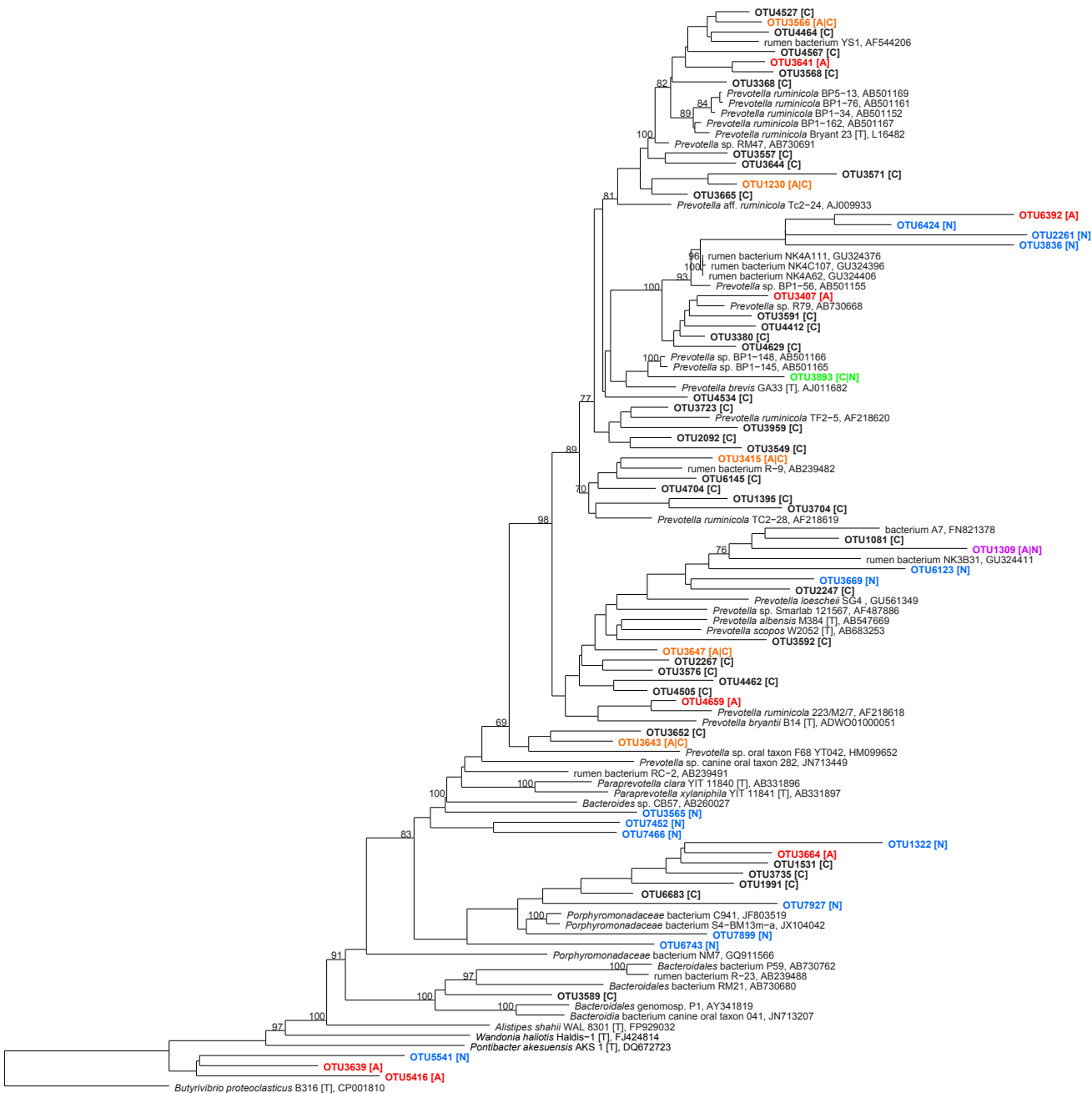

Supplement: Figure S1 — Phylogenetic placement of Bacteroidetes-affiliated novel [N], abundant [(A) Supporting Information Table S5], and core [(C) Supporting Information Table S6] rumen operational taxonomic units (OTUs) in relation to 16S rRNA gene reference sequences of related cultured strains. The phylogenetic tree was inferred from reference sequences using the maximum-likelihood method in combination with a GTRGAMMA substitution model implemented in RAxML. Butyrivibrio proteoclasticus strain B316T (CP001810) was used as an outgroup. Bootstrap values were calculated from 1000 replicates; only values greater than 70% are shown. The scale bar corresponds to the mean number of nucleotide substitutions per site. Novel, abundant and core OTUs were mapped onto these trees using the parsimony insertion tool in arb. [T] indicates a type strain. [file mbt20007-0467-sd1.pdf]

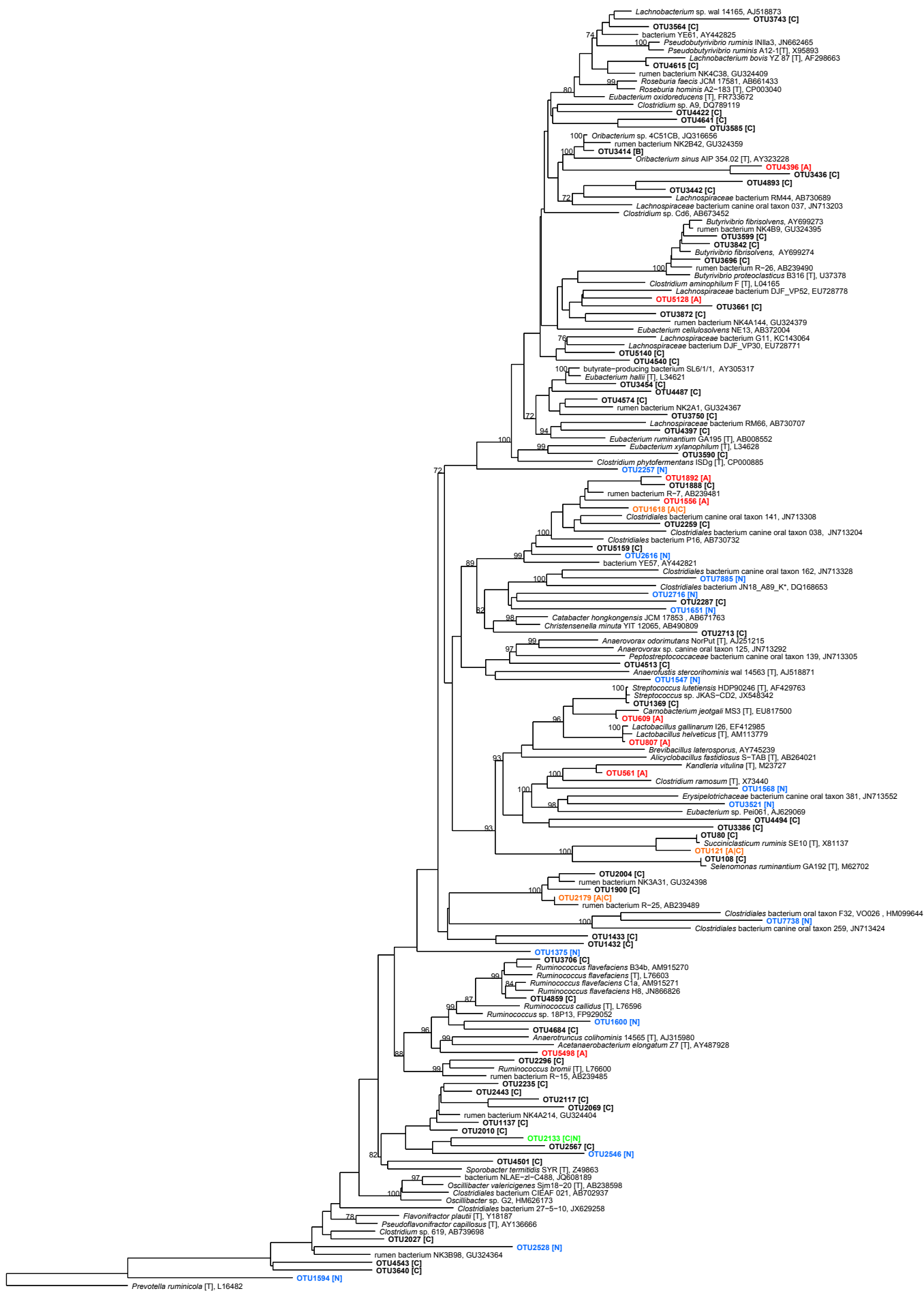

Supplement: Figure S2 — Phylogenetic placement of Firmicutes-affiliated novel [N], abundant [(A) Supporting Information Table S5], and core [(C) Supporting Information Table S6] rumen operational taxonomic units (OTUs) in relation to 16S rRNA gene reference sequences of related cultured strains. The phylogenetic tree was inferred from reference sequences using the maximum-likelihood method in combination with a GTRGAMMA substitution model implemented in RAxML. Prevotella ruminicola strain Bryant 23T (L16482) was used as an outgroup. Bootstrap values were calculated from 1000 replicates; only values greater than 70% are shown. The scale bar corresponds to the mean number of nucleotide substitutions per site. Novel, abundant and core OTUs were mapped onto these trees using the parsimony insertion tool in arb. [T] indicates a type strain. [file mbt20007-0467-sd2.pdf]

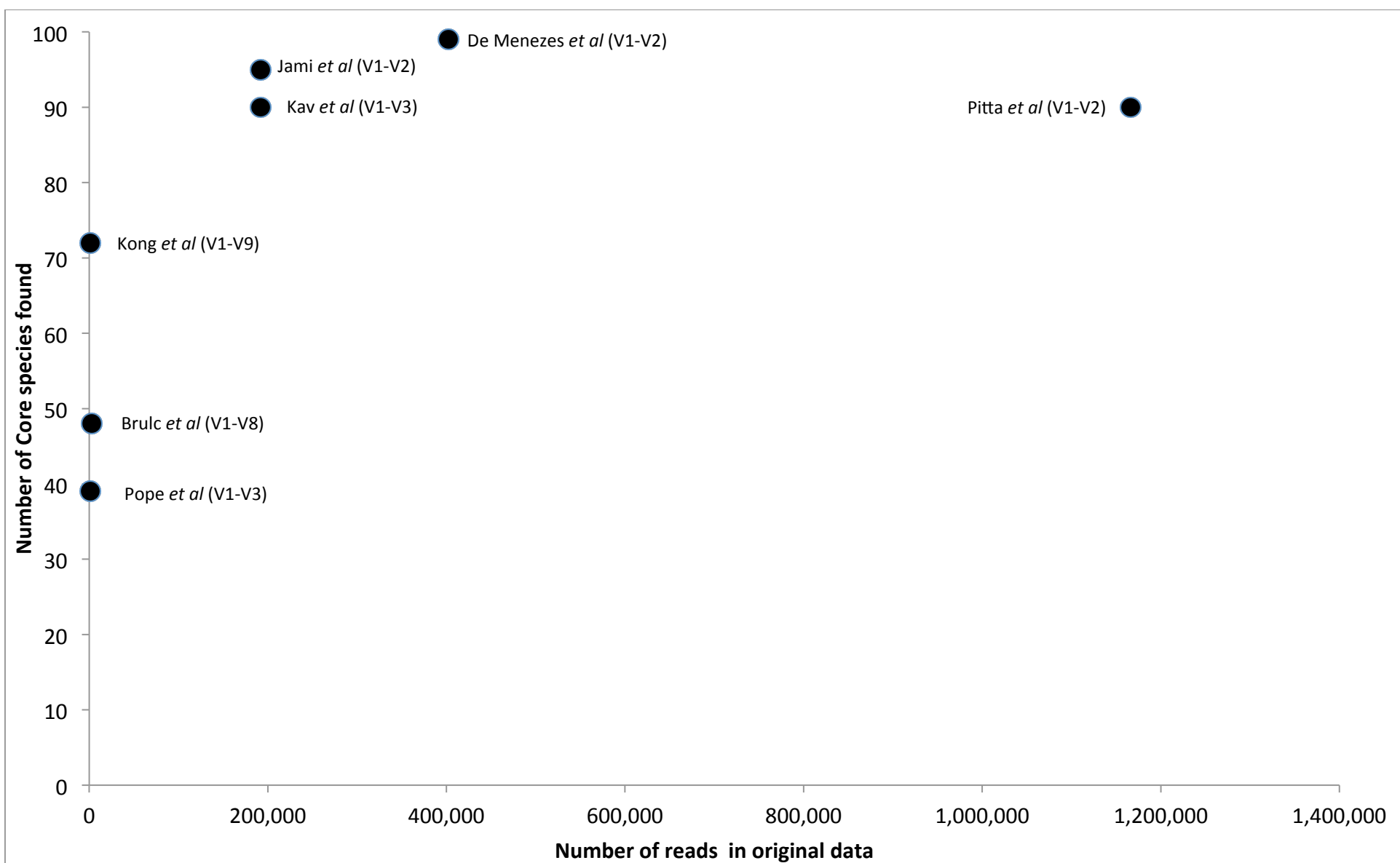

Supplement: Figure S3 — Scatter plot of the number of core OTUs found from each rumen dataset examined, compared with the number of initial reads sequenced. The labels on the points illustrate from which study each was derived and the region of the 16S targeted (see Supporting Information Table S3 for more details). [file mbt20007-0467-sd3.pdf]
